# Unethical behavior and professionalism among medical students in a private medical university in Malaysia

**DOI:** 10.1186/s12909-019-1662-3

**Published:** 2019-06-18

**Authors:** Hematram Yadav, Ravindran Jegasothy, Sowmya Ramakrishnappa, Jaiprakash Mohanraj, Prathapa Senan

**Affiliations:** 1 Department of Community Medicine, Faculty of Medicine, MAHSA University, Selangor, Malaysia; 2 Faculty of Medicine, MAHSA University, Selangor, Malaysia; 3 Department of Biochemistry, Faculty of Medicine, MAHSA University, Selangor, Malaysia

**Keywords:** Professionalism, Ethics, Medical students. Medical education, Discipline, Plagiarism and cheating, Sexual harassment

## Abstract

**Background:**

Ethical behavior and professionalism is an ideal characteristic required of medical students and included as ‘must achieve’ and critical aspect of medical students’ curriculum. This study proposes to determine the perceived unethical and unprofessional behavior among medical students in a private medical university from year 1 to year 5 of the medical curriculum.

**Methods:**

A cross–sectional study was conducted among year 1 to year 5 medical students in a private medical university. A self-administered questionnaire was used with the 3 major domains of professionalism and ethics i.e. discipline plagiarism and cheating.

**Results:**

A total of 464 respondents responded to the survey and they included medical students from year 1 and year 2 (pre-clinical) and years 3–5 (clinical years). Majority of the students, 275 (59.2%) answered that they had not seen any form of unethical behavior among other students. The females seem to have a larger number 172(63%) among the same gender compared to the males. Majority 352 (75%) of them had not heard of the ‘Code of Professional Conduct by the Malaysian Medical Council’. About fifty three (53.1%) of the students answered that the training was sufficient.

**Conclusions:**

This study showed that the perception of unethical behavior was 58.8% in the 1st year (pre-clinical) and it increased to 65.2% in the 5th year (clinical). The 3 main discipline issues were students do not show interest in class (mean 2.9/4), they are rude to other students (mean 2.8/4) and talking during class (mean 2.6/4). Despite the existence of unethical behavior among the students majority of them (71.7%) claimed that they had adequate training in ethics and professionalism. It is proposed that not only the teaching of ethics and professionalism be reviewed but an assessment strategy be introduced to strengthen the importance of professionalism and ethics.

## Background

Ethical behavior and professionalism is an ideal characteristic required of medical students and includes as a ‘must achieve’ and critical aspect of medical students’ curriculum. Professionalism can be defined as a body of qualities or behavior characteristics of a profession and it is an outward visible expression of acceptable behavior of a professional group. It reveals our values, what we stand for, how we behave and perform. American Board of Medical Studies defines medical professionalism as a belief system about how best to organize and deliver health care, which calls on group members to jointly declare (‘profess’) what the public and individual patients can expect regarding shared competency standards and ethical values, and to implement trustworthy means to ensure that all medical professionals live up to these promises [1]. The Malaysian Medical Council does not attempt to define professionalism but outlines a set of behaviors expected of a medical professional [2] and the Malaysian Medical Association (MMA) defines medical ethics as a civil code of behavior considered correct by members of the profession for the good of both the patient and profession. Medical students therefore should respect the rights of patients, colleagues, other health professionals and shall also safeguard the patients’ confidence. This trust goes beyond written words and leads the public at large to expect of the doctor to have not only a high standard of medical ability and skill but also impeccable behavior [3]. It has been shown that medical students’ ethical behaviour does affect their future behaviour while practicing as a medical doctor. In a study of 235 graduates of 3 Medical Schools who had been disciplined by the State Board in United States showed that the residents conduct was strongly associated with prior unprofessional behaviour of the residents while they were in the medical schools [4]. A similar study [5] found that the exposure to unethical and unprofessional behavior is thought to play a major role in the declining empathy experienced by medical students during their training. It has been widely accepted that formal training and professionalism and ethics should be included in the medical curriculum [6–8]. Thus many medical schools lately have made ethics and professionalism an integral part of medical education and training in the last three decades [7] In a preliminary survey of professionalism and teaching practices in Anatomy education in India it was found that faculty members regularly instruct students regarding expected behavior during anatomy course. The instructions include attributes of professionalism like humanism, accountability and honesty [8]. Despite the attempts to train the medical students in professionalism and ethics; it has been shown that the results are not very promising. For instance a study done in Iran among 149 medical interns, clinical residents, and physicians about 44.23% had not heard of the term medical professionalism thus showing that the knowledge of medical professionalism was poor in Iran because professionalism was not taught in the medical students’ curriculum [9]. Similarly it has been found that those students who had witnessed an episode of unethical behavior among other students, were more likely to act improperly themselves for fear of poor evaluation or to fit in with the team [4]. Thus it is important to study the unethical behaviour of the students to see whether the teaching of ethics and professionalism is addressing the ‘right ‘behaviour’ among the students. The aim of this study is to determine the perceived unethical and unprofessional behavior among medical students in a private medical university from year 1 to year 5 of the medical curriculum.

## Methods

A cross–sectional study was conducted among medical students of year 1 to year 5 in a private medical university in Kuala Lumpur, Malaysia. Sample size was calculated using single proportion formula. A minimum sample of 384 was required to determine the unethical behavior and professionalism among medical students. An additional of 10% was added up to the obtained sample size for possible non-response, missing data or errors, thus a final sample size of 423 was required. Convenience sampling method was used to recruit the number of students required considering the limitations of convenience sampling. A self-administered questionnaire was used with 3 major domains of professionalism and ethics i.e. discipline plagiarism and cheating. For discipline there were 7 questions, for plagiarism 3 questions and for sexual harassment we used 2 questions. The questionnaire was validated by doing a pilot study on 40 students (10% of sample size) in the dental faculty of the same university. Reliability analysis for each of the questions was standardized to a Cronbachs alpha value of 0.7. Medical students (Year 1-Year 5) who consented to participate in the study were recruited for the survey. Approval and funding was obtained from the university research committee before commencement of the study. SPSS version 19 was used to analyse that data and *p* < 0.05 was accepted as the significant level.

## Results

A total of 464 respondents completed the survey. The respondents included medical students from year 1 and year 2 (pre-clinical) and year 3–5 (clinical years). Of the total students there were 191 (41.2%) male students and 273 (58.8%) female students. (Table 1). There were 114 (24.6%) students from year 1, 84 (18.1%) from year 2, 101 (21.8%) from year 3, 96(20.7%) from year 4, and 69 (14.9%) from year 5. Majority of the students when asked whether they are aware of any unethical behavior among other students in the University, overall 189 (40.7%) had noticed some form of unethical behavior among other students. Among the males 88 (46.6%) and among the females 101(53.4%) had seen unethical behavior. However the majority of students 275 (59.3%) answered that they have not seen and not aware of any unethical behavior among other students. (Table 1). As for the unethical behavior by years of training it was noticed that in year 1 of study 58.8% were aware of the unethical behavior among students and year 2 it was 46.45, year 3 about 55.4% year 4 it was 70.8% and year 5 it was 65.2%. Overall it was lower in the pre-clinical years (year 1, 2 and 3) and higher in the clinical years (year 4 and 5).

**Table 1:** Awareness of unethical behavior observed among other students in the university by gender

Unethical behavior	Gender		*X*^*2*^ Value	*p*-value
	Male	Female		
No	88 (46.6%)	101 (53.4%)	3.835	0.05
Yes	103 (53.9%)	172 (62.5%)		
Total	191 (41.2%)	273 (58.8%)		

When the students were asked whether they personally experienced any unethical behavior, overall about 168 (36.2%) of them encountered unethical behavior, of these 171(38.1%) were males and 104 (57.1%) were females. However, majority of the students 296 (63.7%) did not encounter any ethical issues during the study in the University (Table 2).

**Table 2:** Personally encountered unethical behavior by Gender

Personally encountered Unethical behavior	Gender		*X*^*2*^ Value	*p*-value
	Male	Female		
No	127 (42.9%)	169 (57.1%)	1.024	0.312
Yes	64 (38.1%)	104 (57.1%)		
Total	191 (41.2%)	273 (58.8%)		

Among the various professionalism values, the three main issues observed among the medical students were discipline, plagiarism and cheating, and sexual harassment (Tables 3). Among the discipline values 333 (71.8%) of the students were found to be rude to other students Students not showing interest in class was 208(44.9%) and absent from class was 259 (55.8%). Plagiarism was seen among 302(65.1%), copying 257 (55.4%), cheating in exams 319 (68.7%) and sexual harassment was noticed among 326 (70.1%) (Table 3).

**Table 3:** Perception of discipline, plagiarism and sexual harassment among medical students

	Disagree	Neutral	Agree	Mean	SD
Discipline				2.54	
Being regularly late to class	136 (29.3%)	328 (70%)	0	2.1	1.4
Rude to other students	85 (18.3%)	46 (9.9%)	333 (71.8%)	2.8	1.3
Does not show interest in class	80 (17.2%)	176 (37.9%)	208 (44.9%)	2.9	1.1
Absent from class regularly	78 (16.8%)	127 (27.4%)	259 (55.8%)	2.6	1.2
Talking in class during lecture	55 (11.9%)	156 (33.6%)	251 (54.1%)	2.6	1.8
Using headphones in class during lecture	81 (17.4%)	175 (37.7%)	208 (44.8%)	2.4	1.1
Sleeping in class during lecture	90 (19.4%)	170 (36.6%)	203 (43.7%)	2.4	1.5
Plagiarism and cheating				2.5	
Plagiarism	73 (15.7%)	88 (19%)	302 (65.1%)	2.6	1.8
Copying assignments form other students	79 (17.0%)	127 (27.4%)	257 (55.4%)	2.6	1.8
Cheating during exams	95 (20.5%)	48 (10.3%)	319 (68.7%)	2.3	2.9
Sexual harassment				2.72	
Sexual harassment from other students	104 (22.5%)	34 (7.3%)	326 (70.1%)	2.9	1.5
Telling vulgar jokes to opposite sex	101 (21.7%)	79 (17.0%)	284 (61.2%)	2.7	1.4

Table 3 shows the perceptions of 3 major domains of professionalism among the students discipline, plagiarism and cheating and sexual harassment. There is a perception that many students do not show interest in class (mean 2.9/4) and rude to other students (mean 2.8/4) and absent from class (mean 2.6/4). Plagiarism and cheating was also high with a mean of 2.6/4 and sexual harassment was also a major problem among the students (mean 2.9/4).

Table 4, About 333 (71.7%) students responded that they had adequate training in the ethics and professionalism and that was provided in the curriculum and only 28.2% responded that they did not have adequate the training. Among the males 131 (68.6%) said that they had adequate training and among the females 202 (74.0%) said that they did not have adequate training. When looked by gender about 60 (31.4%) amongst the males and 71(26%) amongst the females perceived that they did not have adequate training. This could be due to the fact the training is done not as a dedicated module but rather it is incorporated as a professional personal development (PPD) module and the students probably may not realize that it is part of the ethics training. By year of training it was noticed that overall in year 1 MBBS, only 55.3% said that they had some form of training and the percentage was higher in the later years of training, 70.2% in year 2, 74.3% in year 3,79.2% in year 4 and in the final year it was 87.0%.

**Table 4:** Adequate training in ethics and professionalism by gender in all years of training provided in the curriculum

Adequate Ethical training	Gender		*X*^*2*^ Value	*p*- value
	Male	Female		
No	60 (31.4%)	71 (26%)	1.621	0.203
Yes	131 (68.6%)	202 (74.0%)		
Total	191	273		

Majority 352 (75%) of them had not heard of the ‘Code of Professional Conduct by the Malaysian Medical Council’. Amongst the males 139 (72.8%) had not heard of the code and amongst the females 213 (78%) had not heard of the code. Similarly when asked regarding the Code of Medical Ethics by the Malaysian Medical Association majority 361 (78%) had not heard of the ‘Code of Medical Ethics and by gender 146 (76.4%) males and 215 (78.8%) females had not heard of the code. Overall only 103 (22.2%) had heard of the Code of medical Ethics. When it was analyzed by year of study it was found that 14.9% in year 1, 28.6%in year 2, 18.8% in year 3, 20.8% in year 4 and about 46.4% in year 5 which showed that there was slight improvement in the final year.

In Table 5, the students were asked whether the ethical training provided in all the years was sufficient to deal with unethical issues, and 246 (53.1%) answered that the training was sufficient. There was no statistically significant association determined between gender and ethical training as *p*-value = 0.605 (*p* > 0.05).

**Table 5:** Has ethical training so far prepared you to deal with ethical issues and professionalism?

Ethical training	Gender		*X*^*2*^ Value	*p*- value
	Male	Female		
No	104 (54.5%)	142 (52.0%)	0.268	0.605
Yes	87 (45.5%)	131 (48.0%)		
Total	191 (41.2)	273 (58.5%)		

## Discussion

Medical professionalism and ethics is an important aspect of medical education since behavior learnt in medical school affects the behaviour in later years of medical practice. Studies have also shown that preclinical unethical behavior among medical students was associated with professionalism concerns during the clinical years [4, 10]. We studied the three domains of ethics and professionalism which were discipline, plagiarism and cheating and sexual harassment. In our study we found that the overall perception of unethical behavior was 58.8% in the 1st year (pre-clinical) medical students and it increased to 65.2% in the final year (5th year/ clinical). These findings are consistent with a study in the United States which found that medical students’ perception of unethical conduct among residents and attending physicians was about 35% of 1st year students and rose to 90% among 4th year students [11]. These findings were despite the fact that about 53.1% of them had said that professionalism and ethical training in the medical curriculum was sufficient.

The overall mean in the domain of discipline was 2.54/4 and this study showed that students do not show interest in class which had a mean 2.9/4, rude to other students with a mean of 2.8/4 and absent from class with a mean of 2.6/4. These were the main discipline issues perceived by the students. In a similar study lack of punctuality and respect was the most frequently mentioned in a surgical community in Nairobi [12] and another study between two Universities’ physically assaulting another university student and plagiarizing from a fellow students were frequent (76.6%) [13].

In the domain of plagiarism and cheating the overall mean score in our study was 2.5/4 and it was noticed there was high level of cheating during exam (68.7%) and in plagiarism it was 65.1%. Dishonesty and cheating among medical students was also seen in Croatia where 78% of the students admitted frequently cheated in exams [14] and also in Korea where 50 and 78% of the students involved in exam cheating and assignment cheating [15].

In the domain of sexual harassment the overall score was the highest (2.9/4).in our study. About 70.1% had perceived sexual harassment and 61.2% of telling vulgar jokes. However this was also noticed in a study in US where the figures were 83% in women an about 92.8% in men [16]. Similar figures were also observed in several other studies [17, 18] .

This study also showed that despite majority of the students (71.7%) saying that they had `adequate training in ethics and professionalism in the curriculum their level of unethical behavior was still perceived high. It was also surprising to note that majority of the students (75%) had not heard of the Code of Professional Conduct by the Malaysian Medical Council and (78%) had not heard of the Code of Ethics by the Malaysian Medical Association. Unethical behaviour among students is due to one’s personality and it can influence other students differently. A study found about 58% students reported having done something unethical and 61% of those who witnessed unethical behavior by other students’ more than half (54%) felt like they were accomplished to the unethical behaviour [10]. Thus issues of professionalism and ethics is important among any medical school students since they can influence other students’ feelings and emotions. Similarly perception of misbehavior can be perceived differently between the staff and the students and a study found that all faculty members recommended action to be taken against the students for misbehavior but interestingly a study sample of students in the same University suggested ignoring the sanctions [19].

The ethical issues that arise in medical practice are unique and often extremely challenging and should therefore be discussed throughout the course of medical students training [20].There are already efforts to strengthen the ethical and professionalism aspects in our students. The students are now required to take an online medical ethics programme by the World Medical Association in the 4th year of the programme. Teaching methods in the curriculum no longer remain a hidden curriculum but are taught explicitly including use of role models [20, 21]. It is hoped that with the new changes there will be improvement in the future.

## Conclusion

There is very little information about the knowledge on professionalism and ethics training among medical students in Malaysia and this study has shown that there are major weaknesses in the training of ethics and professionalism. It is important to realize that although most medical schools have some form of ethical and professionalism training, assessment and evaluation needs to be done regularly to see whether there is any impact of such training among the students. This is important as unethical behaviour among students is known to affect later years of their practice. We have now made several changes in the training and hope that it will improve in the future.

## Limitation

The definitions of some of the terms were left to the students’ perspective and as such the students may overstate to some questions such as sexual harassment. Although the questions were given to each student but there could be influence from student to student in terms of providing information. Convenience sampling was used and it may cause a bias during data analysis.

## Data Availability

The raw data is the property of the university. However if there is any request it will be provided with permission form the University authorities.
